# A Cross-Sectional Study of Prisoners in Mexico City Comparing Prevalence of Transmissible Infections and Chronic Diseases with That in the General Population

**DOI:** 10.1371/journal.pone.0131718

**Published:** 2015-07-20

**Authors:** Sergio Bautista-Arredondo, Andrea González, Edson Servan-Mori, Fenella Beynon, Luis Juarez-Figueroa, Carlos J. Conde-Glez, Nathalie Gras, Juan Sierra-Madero, Ruy Lopez-Ridaura, Patricia Volkow, Stefano M. Bertozzi

**Affiliations:** 1 National Institute of Public Health, Cuernavaca, Mexico; 2 Mexico City’s AIDS Program, Mexico City, Mexico; 3 Instituto Nacional de Ciencias Médicas y Nutrición Salvador Zubirán, Mexico City, Mexico; 4 National Institute of Oncology, Mexico City, Mexico; 5 University of California, Berkeley, California, United States of America; Kaohsiung Medical University Hospital, Kaohsiung Medical University, TAIWAN

## Abstract

**Objectives:**

To describe patterns of transmissible infections, chronic illnesses, socio-demographic characteristics and risk behaviors in Mexico City prisons, including in comparison to the general population, to identify those currently needing healthcare and inform policy.

**Materials and Methods:**

A cross-sectional study among 17,000 prisoners at 4 Mexico City prisons (June to December 2010). Participation was voluntary, confidential and based on informed consent. Participants were tested for HIV, Hepatitis B & C, syphilis, hypertension, obesity, and, if at risk, glucose and cholesterol. A subset completed a questionnaire on socio-demographic characteristics and risk behaviors. Positive results were delivered with counseling and treatment or referral.

**Results:**

76.8% (15,517/20,196) of men and 92.9% (1,779/1,914) of women participated. Complete data sets were available for 98.8%. The following prevalence data were established for transmissible infections: HIV 0.7%; syphilis: Anti-TP+/VDRL+ 2.0%; Hepatitis B: HBcAb 2.8%, HBsAg 0.15%; Anti-HCV 3.2%. Obesity: 9.5% men, 33.8% women. Compared with national age- and sex-matched data, the relative prevalence was greater for HIV and syphilis among women, HIV and Hepatitis C in men, and all infections in younger participants. Obesity prevalence was similar for women and lower among male participants. The prevalence of previously diagnosed diabetes and hypertension was lower. Questionnaire data (1,934 men, 520 women) demonstrated lower educational levels, increased smoking and substance use compared to national data. High levels of non-sterile tattooing, physical abuse and histories of sexual violence were found.

**Conclusion:**

The study identified that health screening is acceptable to Mexico City prisoners and feasible on a large-scale. It demonstrated higher prevalence of HIV and other infections compared to national data, though low rates compared to international data. Individual participants benefited from earlier diagnosis, treatment and support. The data collected will also enable the formulation of improved policy for this vulnerable group.

## Introduction

Healthcare in prisons is increasingly recognized as a key part of the public health agenda. In the prison population, marginalized groups with increased vulnerability to health problems are over-represented: the economically deprived, the poorly educated and those with substance abuse and other high-risk behaviors. Historically priority has been devoted to custodial duties over care [1]. But from a human rights standpoint, prisoners are entitled to proper healthcare. Inadequate prison healthcare adversely affects the individual as well as the wider community: transmissible diseases can spread within and beyond the prison walls both before release (via prison staff and visitors) and after. Failure to promptly detect and treat prisoners’ health problems leads to higher long-term public health consequences and healthcare costs.

Globally, prison populations have been increasing, leading to overcrowding, which exacerbates the already poor living conditions that can further contribute to health problems. Mexico has one of the largest prison populations in Latin America, with an incarceration rate of 182 per 100,000 [2]. However in Mexico City, according to official sources, the prison population more than doubled from 2003 (17,000 prisoners) to more than 40,000 prisoners in 2010 [3]–a striking local incarceration rate of 426 per 100,000. A survey of the prisons revealed high levels of overcrowding, poor living conditions and poor access to clean water, sanitation and healthcare, as well as corruption [4]. Others have documented similar conditions throughout Latin America [5].

Many studies internationally have documented the significantly increased prevalence of transmissible infections in prisons [2,6–8]. Before this study, little was known about the prevalence of transmissible infections in Mexican prisoners. In 1998 a comparison of data from the penal system and national HIV program found increased prevalence in prisons in 13 of the 14 states studied [9]. Later, in Durango, prison prevalence of Hepatitis A, B, D and HIV was found to be comparable to that of the general population, though HCV prevalence was higher than that of blood-bank donors [10].

In Mexico, there is no routine testing for transmissible infections on entry to prison. HIV testing has only been done when prison health staff suspects infection. Before the start of the study, about 80 prisoners in Mexico City were known to have HIV, i.e., about 0.2% of the prison population compared to 0.3% nationally, and under-diagnosis was suspected. Information about other infections was lacking.

There are very few studies internationally on the prevalence of chronic diseases in prisons. Some recent findings from prisons in the USA have demonstrated levels of obesity and chronic diseases comparable to those of the general population [11]; others have found higher levels among prisoners [12]. In Australia, 81% of female prisoners and 65% of male reported at least one chronic health problem [13]. However female prisoners in Japan showed a decrease of blood pressure and dyslipidemia probably related to enforced lifestyle changes in prison [14].

There are no studies of chronic health problems among Mexican prisoners, but, given the high prevalence of obesity, diabetes and related morbidity in the Mexican population, this is a key area to address. Diabetes, ischemic heart disease and cerebrovascular disease are leading causes of death in Mexico, accounting for 186,158 (31.4% of total) deaths in 2010 [15].

In order to devise appropriate policies to tackle health problems in prisons and avert adverse effects on the wider community, reliable data must be available. Up to now there has been no systematic health screening in Mexico City prisons, and little research into risk behaviors that may contribute to prisoners’ health problems in the context of low- and middle-income countries. In this context, this study is unique in its scope, offering the entire population of four Mexico City prisons (>22,000 individuals) screening for infections: HIV, Hepatitis B (HBV), Hepatitis C (HCV) and syphilis; and four chronic health problems: hypertension, diabetes, obesity and dyslipidemia. The overarching goal of the project was to document the prevalence rates of infectious disease and key health problems of the prison population in Mexico City and understand the differences in health problems between prisoners and the general population. In identifying these health problems, the aim was to increase health awareness and provide access to healthcare services for those in need. Furthermore, our expectation is that the results can be used to prioritize, design and implement future prison health interventions—both prevention and treatment—in a cost-effective and holistic manner.

## Materials and Methods

This cross-sectional study was carried out in two male and two female prisons in Mexico City from June to December 2010. It consisted of two main components: a confidential general health assessment for all participants covering HIV, HBV, HCV and syphilis, obesity, hypertension and, for those considered at risk, diabetes and dyslipidemia; and an anonymous self-completed questionnaire for a representative sample on determinants and associated risk factors with the themes in the health assessment section. The project was reviewed and approved by the Committees of Research, Biosafety and Ethics of the INSP, Mexico. (Research committee: 821 N°712, Biosecurity committee: 813).

Before the start of the study, a meeting was held with the relevant authorities of each prison to reach a consensus about participation and logistical issues. A pilot study was carried out in a small center (about 400 prisoners) after which some adjustments were made, and the final strategy is described below. An awareness campaign where the message was “put yourself to the test” consisting of leaflets, posters and videos was disseminated before the start of the study and throughout the implementation period. The campaign provided comprehensive information on the study’s objectives and methods, the benefits of participation, and a full description of all health problems covered by the study: causes, symptoms, diagnosis, and treatment options. The objectives were to improve the quality of the informed consent process and to increase participation rates.

To participate in the study, all prisoners were invited, in groups of 10, to attend an informational session about the study, during which the consent form was read aloud and given to each inmate to read. Trained staff answered any concerns individually, and prisoners could opt to take information away and participate later if they wished. In line with international guidance for research involving prisoners, participation was completely voluntary; participants received no monetary compensation, but were given a small pack containing toiletries as a token of appreciation. No additional incentives were given for completing the questionnaire.

Those who agreed to participate were allocated a unique identifier number and were assessed as detailed below. All the information collected remained confidential—only those delivering results could access names, and only the analytical team was able to view questionnaire results.

### 2.1 Health assessment

#### Measurements

Blood pressure (BP) was measured in both arms using an electronic sphygmomanometer; this was repeated with a minimum 2-minute interval, and then all readings averaged to give a mean systolic / diastolic BP. Waist circumference, weight and height were measured twice (shoes, belts, items in pockets were removed for the measurement), and averaged. Body Mass Index (BMI) was calculated and categorized as WHO standards (underweight <18.5 kg/m2, normal 18.5-<25 kg/m2, overweight≥25.0-<30 kg/m2 and obese≥30 kg/m2)[16].

#### Screening questions

The answers to the screening questions for diabetes, hypertension and hyperlipidemia were recorded by trained fieldwork staff and entered onto a computer database, along with the BP and anthropometric data already collected. A software algorithm recognized those with elevated risk of diabetes and dyslipidemia to prompt additional testing (of glucose and cholesterol). To trigger random glucose and cholesterol testing, participants needed one or more of the following criteria: BMI≥30 kg/m2; BP≥140/90mmHg; ≥10 points in the validated risk factor questionnaire used in Mexico, adapted from the ADA [17].

#### Blood samples

All participants had a sample taken to test for HIV, HBV, HCV and syphil**is**. HIV 1 and 2 antibodies and p24 antigen were tested using Abbott Architect Ag/Ab Combo. Hepatitis B core antibody (HBcAb) was tested using Abbott Architect Anti-HBc II. If this was positive, the sample was tested for Hepatitis B surface antigen (HBsAg). Hepatitis C Antibodies were tested using Abbott Architect Anti-HCV. Treponema pallidum antibody (Anti-TP) was tested using Abbott Architect Syphilis TP; if this was positive, Venereal Disease Research Laboratory VDRL was done. Participants were considered to have syphilis requiring treatment if both tests were positive.

Each of the initial tests above was completed using Chemiluminescent *Immunoassay* (CLIA) in pools of 4 participant samples. If a pool was positive, each sample was tested individually. This reduced the cost without reducing the sensitivity, as reported previously [18,19].

In addition, those identified as at risk of diabetes or hypercholesterolemia had samples taken for random blood glucose and lipid profile.

#### Recall

Once initial results were available, those participants who had an abnormal random blood glucose (≥140mg/dL) or cholesterol (≥200mg/dL), or had a previous diagnosis of diabetes, dyslipidemia or hypertension, were asked to return for a fasting test of glucose and lipid profile. HbA1c was not available to test diabetic control for those already diagnosed with diabetes due to funding, and therefore fasting glucose≥140 mg/dl was used as a marker of uncontrolled diabetes. The second test visit was on average two weeks after the initial assessment.

Abnormal results for chronic diseases (among participants without previous diagnoses) were considered in the following categories: fasting glucose≥126mg/dl—probable diabetes; fasting glucose≥100 and <126 mg/dl—probable pre-diabetes; blood pressure≥140/90 mmHg—probable hypertension; blood pressure≥130/85 and <140/90 –probable pre-hypertension; fasting low density lipoprotein cholesterol (LDLc)≥100mg/dl—probable hyperlipoproteinemia.

#### Delivering results

Individual printed results summaries were given to all participants, along with prevention information related to the health aspects addressed by the study. Participants who did not return for their results were called on each occasion that the fieldwork team was in that center. All participants were given contact details for the team leads in case they were released before receiving results. Participants with any positive result(s) received counseling by trained health professionals that included an explanation of their health problem, **a**nd advice for follow-up and treatment. Post-test counseling and direct referral with consent to an HIV specialist were carried out for those found to be HIV positive. Those positive for both Anti-TP+/VDRL+ were assumed to have syphilis requiring treatment. This was explained and participants were treated with intramuscular benzathine penicillin (1,200,000 IUx2 doses) at the time of results delivery and were referred for 2 further weekly doses. Those with positive results for any other problem were referred to the general prison health service and given lifestyle advice and information about preventing transmission or complications. Information on theirs subsequent care was therefore not available.

### 2.2 Questionnaire

A representative subset of individuals who had completed the initial health assessment were selected by simple random sampling procedure with replacemen**t**; about 15% men and 30% women (a greater percentage of women were selected due to the lower total number of female participants), was randomly selected to complete an anonymous questionnaire at the end of the initial health assessment, using the Audio Computer-Assisted Self-Interview method [20]. A program was designed to read out the questions and options for answers to allow those with literacy difficulties to complete the questionnaire easily and privately. However if the participant requested, support staff were available to help. The questionnaire included detailed questions on socio-demographic data, substance abuse, crime history, sexual behavior, diet, physical activity, psycho-social and (for women only) reproductive histories.

### 2.3 National Health and Nutrition Survey 2006

Unpublished epidemiological data from the Encuesta Nacional de Salud y Nutricion—National Health and Nutrition Survey (ENSANUT) 2006 was analyzed to compare results from prisons with the general population. The objective of ENSANUT 2006 was to update the prevalence of infectious and chronic diseases and their associated risk factors among a nationally representative sample of the general population in Mexico. This survey collected health and nutrition data from October 2005 to May 2006 using a probabilistic, multistage, stratified cluster sampling design. In addition to be nationally representative, the survey is representative at the state level, at regional level (for four geographical regions), and at the rural and urban level.[21] A total of 47,152 households were visited. Among a nationally representative sub-sample of adults (20 years and older), blood pressure, glucose, insulin, triglycerides, total cholesterol, and HDL-cholesterol and anthropometric measurements were obtained.[22] In order to estimate prevalence of sexually transmitted infections, sera were collected from consenting participating adult subjects throughout Mexico, samples were kept frozen until analyzed at INSP laboratory by the same diagnostic techniques employed in this study.

### 2.4 Data analysis

Analyses of the results from the health assessment and questionnaire were carried out on participants with a complete set of basic data available—including age, BMI, BP, and results of tests for infectious diseases. Analyses were performed with Stata/SE 13.1 software **[23]**.

## Results

At the time of the study 22,090 prisoners were registered at the 4 prisons involved, of which 20,176 (91.3%) were men. 15,517 (77.2%) men and 1,779 (92.9%) women consented to participate. Analysis was carried out on 15,354 (98.9%) men and 1,730 (97.2%) women who had a complete set of basic data from the health assessment and 1,934 men and 520 women who accepted and answered the questionnaire. The mean age of male participants was 33.1 (SD 9.3, range 18–81) and female participants 34.9 (SD 10.2, range 18–77).

### 3.1 Study prevalence of transmissible infections and non-communicable conditions

#### Transmissible infections

A total of 105 (0.7%) men and 13 (0.8%) women were found to be HIV positive. In the case of men, this prevalence represents only newly diagnosed cases of HIV given that the 80 already known cases across the whole Mexico City prison system (40,000 prisoners) were concentrated in a single facility, not included in this study, where antiretroviral treatment and HIV care is provided (and therefore the system-wide prevalence for men is likely higher). HCV antibodies were found in 3.3% of the men and 2.6% of the women. HBcAb and HBsAg were found in 2.8% and 0.1% of the men respectively and 3.0% and 0.3% of the women. Anti-TP+/VDRL+ were positive in 1.8% of the men and 3.3% of the women. Table 1 displays prevalence of these infections by sex and age, including comparisons with national data.

**Table 1:** Prevalence of transmissible infections by sex and age.

	Hepatitis B		Hepatitis C Anti-HCV	Syphilis		HIV
	Hep B core Ab + Hep B ‘s’ Ag -	Hep B core Ab + Hep B ‘s’ Ag +*		Anti-TP Ab + VDRL-	Anti-TP Ab+ VDRL+	
	**Prevalence (%) [CI95%]**					
Overall (N = 17,084)	2.83 [2.59;3.09]	0.15 [0.10;0.22]	3.21 [2.95;3.48]	2.12 [1.92;2.35]	1.98 [1.79;2.20]	0.69 [0.58;0.83]
Women (N = 1,730)	2.95 [2.24;3.86]	0.29 [0.10;0.70]	2.60 [1.94;3.47]	5.66 [4.67;6.86]	3.29 [2.55;4.25]	0.75 [0.43;1.30]
Men (N = 15,354)	2.82 [2.57;3.09]	0.14 [0.09;0.21]	3.28 [3.01;3.57]	1.73 [1.53;1.94]	1.84 [1.64;2.06]	0.68 [0.56;0.83]
<30 yrs.						
Women (N = 599)	2.34 [1.36;3.92]	ncr	0.50 [0.10;1.53]	5.34 [3.79;7.47]	3.34 [2.14;5.13]	1.34 [0.63;2.66]
Men (N = 6,256)	1.44 [1.17;1.77]	0.06 [0.02;0.17]	0.85 [0.65;1.11]	0.78 [0.59;1.04]	1.87 [1.56;2.24]	0.72 [0.54;0.96]
30–39 yrs.						
Women (N = 609)	2.63 [1.59;4.26]	0.16 [0.00;1.02]	2.79 [1.72;4.46]	5.42 [3.86;7.53]	3.78 [2.50;5.63]	0.66 [0.19;1.74]
Men (N = 5,659)	3.34 [2.90;3.84]	0.16 [0.08;0.31]	3.98 [3.50;4.52]	1.33 [1.06;1.66]	1.73 [1.42;2.11]	0.88 [0.67;1.17]
≥40 yrs.						
Women (N = 522)	4.02 [2.61;6.10]	0.77 [0.22;2.03]	4.79 [3.24;7.00]	6.32 [4.51;8.77]	2.68 [1.56;4.49]	0.19 [0.00;1.19]
Men (N = 3,439)	4.48 [3.83;5.22]	0.23 [0.11;0.47]	6.54 [5.76;7.42]	4.10 [3.48;4.82]	1.95 [1.53;2.47]	0.29 [0.15;0.54]
Age adjusted OR’s (prison = 1, general population = 0)						
Women	na	0.38 [0.15;0.98]	6.65 [4.37;10.1]	na	2.41 [1.60;3.64]	na**
Men	na	0.16 [0.10;0.26]	6.17 [4.48;8.51]	na	1.71 [1.30;2.25]	2.82 [1.48;5.38]

Note: ncr: no cases reported. na: not applicable.

* In general population: HBV core Ab +.

** Insufficient sample size.

#### Chronic non-communicable diseases

**Fig 1:**
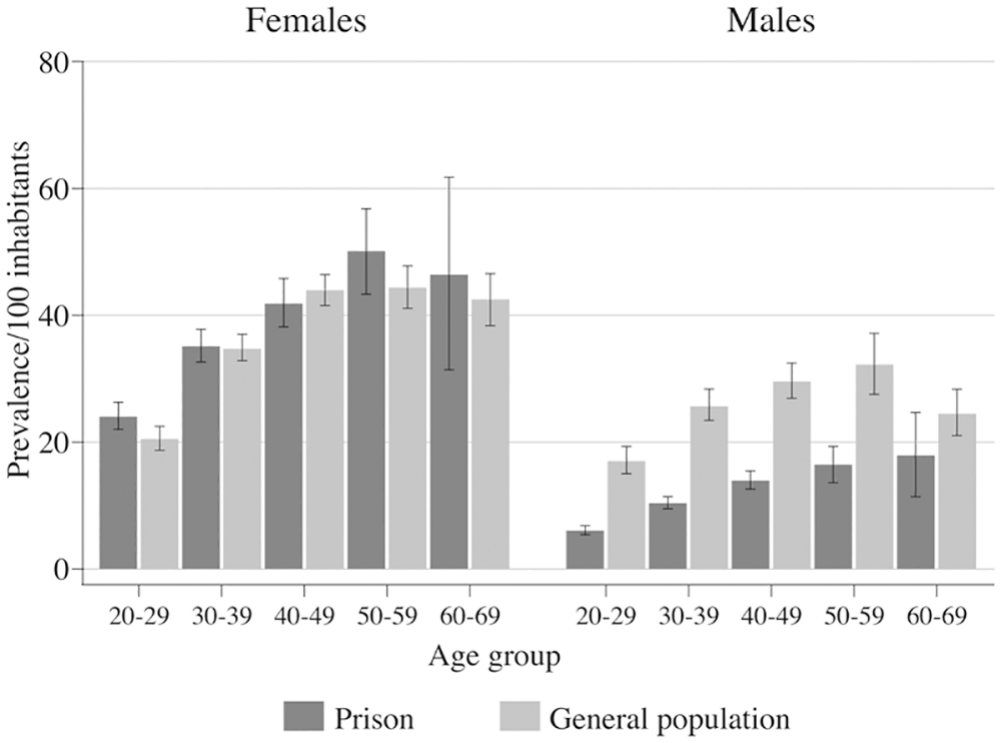
Prevalence of obesity in Mexican prisoners and general population.

Obesity: A total of 9.5% of the men and 33.8% of the women were obese (BMI≥30), 32.4% of the men and 39.7% of the women were overweight (BMI y: A total, 56.28% of the men and 25.6% of the women had a BMI within the normal range (BMI .28% of the men and 25.6% of the women had a BMI within the normal eight (BMI<18.5). Fig 1 shows prevalence by sex and age and the comparison with national data.

Hypertension: 2.5% of the men and 14.8% of the women reported a previous diagnosis of hypertension (Table 2), of which 39.6% men and 40.6% women were taking medication. Of those (taking and not taking medication), 47.7% and 24.7% of the men respectively and 41.2% and 9.4% of the women had uncontrolled hypertension (BP≥140/90). 3.8% of the men and 2.9% of the women were found to have probable new hypertension (BP≥140/90) and 11.6% of the men and 9.4% of the women probable pre-hypertension (BP≥130/85). The highest prevalence of probable new HTN (BP≥140/90) diagnoses was seen in the 60–69 age group for men (40.0%) and women (42.9%).

**Table 2:** Hypertension and diabetes: prevalence and control in the study population.

	Overall (N = 17,084)		<30 yrs.		30–39 yrs.		≥40 yrs.	
	Women (n = 1,730)	Men (n = 15,354)	Women (n = 599)	Men (n = 6,256)	Women (n = 609)	Men (n = 5,659)	Women (n = 522)	Men (n = 3,439)
	**Prevalence (%) [CI95%]**							
Hypertension								
Previous diagnosis of HTN	14.8 [13.2;16.6]	2.51 [2.27;2.77]	9.35 [7.26;12.0]	1.02 [0.80;1.31]	9.85 [7.72;12.5]	1.84 [1.52;2.22]	26.8 [23.2;30.8]	6.31 [5.54;7.17]
Taking medication	40.2 [34.4;46.3]	40.0 [35.2;45.0]	14.3 [7.16;26.0]	18.8 [10.9;30.1]	23.3 [14.3;35.6]	25.0 [17.6;34.2]	57.9 [49.6;65.7]	53.5 [46.8;60.0]
and BP ≥ 140 /90	40.8 [31.8;50.4]	48.7 [40.9;56.5]	ncr	16.7 [3.50;46.0]	50.0 [26.8;73.2]	19.2 [8.05;38.3]	43.2 [33.0;54.1]	58.6 [49.5;67.2]
Not taking medication	59.8 [53.7;65.6]	60.0 [55.0;64.8]	85.7 [74.0;92.8]	81.3 [69.9;89.1]	76.7 [64.4;85.7]	75.0 [65.8;82.4]	42.1 [34.3;50.4]	46.5 [40.0;53.2]
and BP ≥ 140 /90	9.15 [5.42;14.9]	25.1 [19.9;31.1]	ncr	13.5 [6.37;25.6]	6.52 [1.59;18.2]	16.7 [9.87;26.6]	18.6 [10.6;30.6]	37.6 [28.8;47.4]
Fasting LDL ≥ 100 (%)	55.2 [46.1;63.9]	63.6 [55.9;70.6]	50.0 [23.7;76.3]	30.8 [12.4;58.0]	37.5 [18.4;61.5]	53.3 [36.1;69.8]	58.9 [48.6;68.5]	69.7 [61.0;77.3]
Probable new HTN—BP ≥ 140 / 90	3.32 [2.52;4.38]	3.86 [3.56;4.18]	0.18 [0.00;1.15]	2.02 [1.70;2.40]	2.00 [1.08;3.60]	2.66 [2.27;3.12]	9.69 [7.08;13.1]	9.47 [8.50;10.5]
Probable new pre-HTN BP ≥ 130 / 85	4.63 [3.65;5.86]	9.67 [9.19;10.2]	2.58 [1.51;4.33]	8.64 [7.96;9.37]	4.46 [2.99;6.58]	8.56 [7.85;9.34]	8.12 [5.64;11.5]	13.8 [12.6;15.2]
Diabetes								
Previous diagnosis of diabetes	4.62 [3.73;5.72]	1.77 [1.57;1.99]	1.17 [0.52;2.44]	0.21 [0.12;0.36]	2.13 [1.21;3.65]	1.25 [0.99;1.58]	11.5 [9.02;14.5]	5.44 [4.73;6.25]
Taking medication for diabetes	78.8 [68.5;86.4]	70.8 [65.2;75.9]	14.3 [0.53;53.3]	53.8 [29.1;76.8]	84.6 [56.5;96.9]	59.2 [47.5;69.8]	85.0 [73.7;92.1]	76.5 [69.9;82.0]
Fasting glucose result: Glucose ≥ 140 mg/dl	59.3 [46.6;70.9]	62.1 [54.6;69.1]	100	25.0 [3.41;71.1]	70.0 [39.2;89.7]	61.1 [44.8;75.2]	56.3 [42.3;69.3]	63.6 [55.0;71.4]
Not taking medication for diabetes	21.3 [13.6;31.5]	29.2 [24.1;34.8]	85.7 [46.7;99.5]	46.2 [23.2;70.9]	15.4 [3.10;43.5]	40.8 [30.2;52.5]	15.0 [7.87;26.3]	23.5 [18.0;30.1]
Fasting glucose result: Glucose ≥ 140 mg/dl	71.4 [35.2;92.4]	33.3 [23.1;45.4]	100	ncr	ncr	41.7 [24.4;61.2]	66.7 [29.6;90.7]	32.4 [19.5;48.6]
Uncontrolled hypertension (BP ≥ 130/80mmHg)	38.8 [28.8;49.7]	44.3 [38.5;50.2]	14.3 [0.53;53.3]	15.4 [3.10;43.5]	23.1 [7.50;50.9]	31.0 [21.4;42.5]	45.0 [33.1;57.5]	51.3 [44.2;58.4]
Fasting LDLc result: Abnormal LDL cholesterol (LDLc ≥ 100mg/dl)	59.1 [47.0;70.1]	59.1 [52.8;65.2]	100	11.1 [0.00;45.7]	60.0 [31.2;83.3]	58.3 [45.7;69.9]	57.4 [44.2;69.7]	62.0 [54.5;69.1]
Probable diabetes previously undiagnosed (fasting glucose ≥126mg/dl)	16.3 [11.4;22.7]	6.34 [4.89;8.18]	ncr	0.36 [0.00;2.20]	22.0 [11.8;36.9]	2.08 [0.85;4.58]	16.7 [10.7;24.9]	16.1 [12.3;20.7]
Probable Pre-diabetes (fasting glucose 100-125mg/dl)	27.7 [21.4;35.0]	15.5 [13.2;18.0]	ncr	6.07 [3.76;9.57]	26.8 [15.6;42.1]	14.2 [10.6;18.8]	32.4 [24.3;41.7]	25.4 [20.8;30.7]

Note: ncr: no cases reported.

Diabetes: 1.8% of the men and 4.6% of the women reported a previous diagnosis of diabetes (Table 2), of which 70.7% of the men and 78.2% of the women were taking medication, and of them, 62.5% men and 60.3% women who attended for a fasting glucose test had poor control (fasting glucose≥140 mg/dl). Of those with previously diagnosed diabetes, 59.0% of the men and 58.5% of the women who returned for a fasting blood test had abnormally high low-density cholesterol (LDLc≥100mg/dl) and 44.4% of men and 37.2% of women had uncontrolled hypertension (BP≥130/80mmHg).

0.4% of the men and 1.6% of the women had probable previously undiagnosed diabetes (fasting glucose≥126mg/dl) and 0.9% of men and 2.7% of women probable previously undiagnosed pre-diabetes (fasting glucose 100-125mg/dl). The prevalence of probable previously undiagnosed diabetes was highest in the 50–59 age group for women (9.1%) and 60–69 group for men (6.9%).

### 3.2 Comparison with the general population

**Fig 2:**
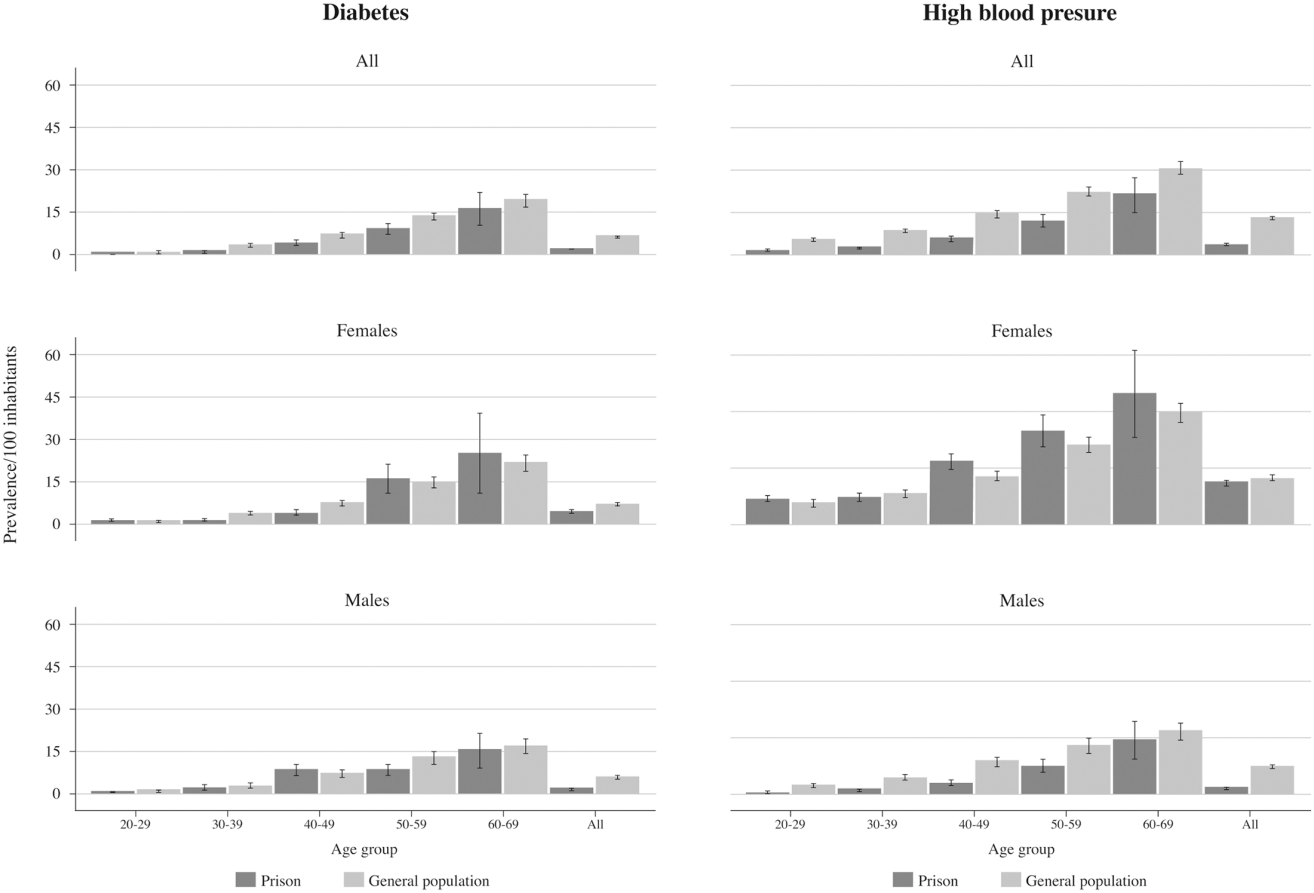
Prevalence of previously diagnosed diabetes and high blood presure in Mexican prisoners and general population.

In order to compare the prevalence data found in the study with a national data subsample we estimated age-adjusted Logit regressions to estimate odds ratios. With the exception of HBV, prison populations had significant higher odds to have a transmissible infection than the general population (Table 1). The odds are particularly high for HCV with the prison population being 6 times more likely to be infected than the general population, however the odds are also higher in the case of syphilis and HIV. The prevalence of obesity was similar between female participants and their national counterparts, but lower among men in the prisons compared with the men in the general population (Fig 1). Previous diagnoses of hypertension and diabetes increased with age, and were lower than the general population, though this varied with age and sex (Fig 2).

### 3.3 Socio-demographic and behavioral risk factors

Analysis was performed on a total of 1,934 (12.5%) men and 520 (29.2%) women who responded to the questionnaire with a corresponding valid identification number from the health assessment. Average age for men was 33.8 years (SD 9.4, range 18–77) and women 34.9 years (SD 10.1, range 18–69).

#### Education

71.4% of men and 65.2% of women completed less than 10 years of formal education. Nationally, for comparison, 48.3% of men and 50.8% of women have less than 10 years formal education [15]. Differences were most marked in the lower percentage of men that had enrolled in university or similar studies (7.2% study population, 25.7% nationally vs. women 16.7% study, 20.7% nationally).

#### Smoking

Reported cigarette smoking was highly prevalent: 61.5% (981) men and 53.8% (225) women reported they were current smokers, compared to 24.8% men and 7.8% women nationally [24]. 18.6% (297) men and 12.4% (52) women in the study population were ex-smokers and 19.9% (317) and 33.7% (141) reported they never smoked; nationally 54.1% men and 83.4% women have never smoked.

#### Drug use

Reported use of illicit substances was highly prevalent among participants. 69.9% men and 59.1% women reported having used drugs at some point: Table 3 details reported drug use by type, and use before and during imprisonment. 5.4% men and 3.7% women reported having injected drugs at least once, of which 26.3% men and 30.8% women reported having shared needles/syringes at least once and 8.3% men and 2.5% women reported sharing needles at least once in prison. 48.4% men and 55.1% women reported injecting non-prescribed vitamins, of which 2.3% men and 0% women had shared injecting equipment ever outside the prison and 3.3% men and 5.4% women had shared syringes last month during incarceration. Of those who reported injecting drug use, 16.3% men and 13.3% women reported not having injected drugs during the 6 months prior to last incarceration.

**Table 3:** Reported drug use by type, and use before and during imprisonment.

**Lifetime use**	**Men; % (N° of cases)**		**Women; % (N° of cases)**	
Any illicit drug	69.9 (1,193)		59.1 (211)	
Marijuana	55.7 (950)		40.9 (146)	
Solvents	28.8 (491)		20.7 (74)	
Cocaine / Crack	43.9 (749)		38.1 (136)	
Pills	22.9 (391)		28.9 (103)	
Heroin	2.5 (42)		1.7 (6)	
Injected drugs	5.4 (102)		3.7 (18)	
Shared syringes before prison (ever)*	26.3 (20)		30.8 (4)	
Shared syringes in prison (ever)*	8.3 (4)		2.5 (3)	
Injected vitamins	48.4 (914)		55.1 (271)	
Shared syringes before prison (ever)*	2.3 (13)		ncr	
Shared syringes in prison (last month)*	3.3 (6)		5.4 (2)	
**Six months before prison / last month in prison**	**Before prison; % of lifetime users (N° of cases)**	**In prison; % of lifetime users (N° of cases)**	**Before prison; % of lifetime users (N° of cases)**	**In prison; % of lifetime users (N° of cases)**
Marijuana				
Every day	35.4 (332)	38.3 (355)	25.7 (37)	26.9 (39)
Three times a week	10.6 (99)	11.3 (105)	7.6 (11)	9.0 (13)
Never used out of prison	16.2 (152)	—	19.4 (28)	—
Never used in prison	—	23.5 (218)	—	30.3 (44)
Solvents				
Every day	14.0 (68)	4.4 (21)	16.4 (12)	1.4 (1)
Three times a week	11.5 (56)	6.6 (32)	13.7 (12)	ncr
Never used out of prison	17.5 (85)	—	6.8 (5)	—
Never used in prison	—	46.1 (222)	—	65.3 (47)
Cocaine / Crack				
Every day	27.9 (204)	6.6 (48)	48.5 (65)	7.6 (10)
Three times a week	11.8 (86)	4.4 (32)	11.2 (15)	4.5 (6)
Never used out of prison	10.5 (77)	—	9.7 (13)	—
Never used in prison	—	48.5 (353)	—	49.2 (65)
Pills				
Every day	16.4 (63)	5.2 (20)	18.6 (19)	6.0 (6)
Three times a week	8.9 (34)	3.1 (12)	5.9 (6)	1.0 (1)
Never used out of prison	17.7 (68)	—	32.4 (33)	—
Never used in prison	—	50.1 (191)	—	40.0 (40)
Injected drugs				
Every day	25.0 (23)	4.5 (4)	6.7 (1)	ncr
Three times a week	3.3 (3)	3.4 (3)	13.3 (2)	ncr
Never used out of prison	16.3 (15)	—	13.3 (2)	—
Never used in prison	—	57.6 (53)	—	50.0 (9)
Injected vitamins				
Every day	4.1 (36)	2.5 (22)	1.1 (3)	0.4 (1)
Three times a week	5.5 (49)	3.7 (32)	4.9 (13)	3.9 (10)
Never used out of prison	8.9 (79)	—	13.7 (36)	—
Never used in prison	—	40.8 (353)	—	33.6 (87)

Note:

* Out-off valid cases (excluding missing or prefer not to answer). ncr: no cases reported.

#### Tattoos

55.5% men and 47.3% women had at least one tattoo. Of those, 52.0% of men and 40.9% of women had received a tattoo in prison. 47.3% of men and 37.6% of women responded that they had knowingly used unsterile equipment for at least one of their tattoos outside the prison.

#### Physical and sexual violence

26.8% of men and 23.5% of women were victims of physical violence whilst incarcerated. Response rates to questions about sexual violence were low. Of those who responded, 0.2% men and 1.3% women reported being a victim of sexual violence during incarceration, while 3.0% men and 21.9% women reported being a victim prior to incarceration. Having been a perpetrator of physical or sexual violence during incarceration was less frequent than those who reported having been a perpetrator prior to incarceration. Table 4 displays prevalence of physical and sexual violence and sexual risk behavior before and during imprisonment.

**Table 4:** Prevalence of physical and sexual violence and sexual risk behavior before and during imprisonment.

	In prison		Before prison	
	Men; % (N° of cases)	Women; % (N° of cases)	Men; % (N° of cases)	Women; % (N° of cases)
Physical violence				
Victim	26.8 (495)	23.5 (110)	na	na
Offender	15.8 (292)	10.2 (49)	18.7 (346)	14.1 (67)
Sexual violence				
Victim	0.2 (1)	1.3 (2)	3.0 (15)	21.9 (25)
Offender	0.3 (2)	1.3 (2)	2.8 (14)	4.4 (5)
Sexual risk behaviour				
Used condom at last sexual encounter	20.5 (120)	18.3 (21)	20.9 (196)	16.9 (55)
Used alcohol/drugs at last sexual encounter	4.1 (26)	8.1 (12)	19.1 (195)	21.4 (81)
Transactional sex				
Gave money/favor for sex	2.0 (13)	2.6 (4)	22.1 (112)	0.9 (1)
Received money/favor for sex	0.6 (4)	2.6 (4)	8.9 (45)	18.8 (21)

Note: na: not applicable.

#### Sexual risk behavior

Condom use was low, but similar in and out of prison– 20.5% and 20.9% of the men and 18.3% and 16.9% of the women used a condom during their last sexual encounter (Table 4). Drug/alcohol use during sexual encounters was reported to be lower during imprisonment compared to prior to incarceration– 4.1% vs. 19.1% of the men and 8.1% vs. 21.4% of the women. 8.9% of the men and 18.8% of the women out of prison and 0.6% of the men and 2.6% of the women in prison reported receiving money or favors for sex. 22.1% of the men and 0.9% of the women out of prison and 2.0% men and 2.6% women in prison reported giving money or favors for sex.

#### Depression

Symptoms of depression were graded using the Beck depression inventory [25]. 23.5% of the men and 23.1% of the women reported mild depressive symptoms, 13.0% and 16.7% reported moderate symptoms and 2.0% and 3.7% reported severe symptoms, respectively.

#### Physical activity

40.2% men and 31.0% women reported an increase in physical activity since incarceration, 21.3% and 20.2% the same and 38.5% and 48.9% less, respectively, for men and women.

## Discussion

Prisoners represent a significant yet previously neglected study population in Mexico City. This study has led to a more comprehensive understanding of the health and socio-demographic profile of prisoners in Mexico City and has already led to improved health awareness among prisoners and prison staff and to the implementation of new care strategies. The study demonstrated that health screening is both acceptable to the great majority of the population (77% men and 93% women participated) and is logistically feasible on a large-scale. The study identified a vulnerable population in socio-economic and risk behaviors and a significant number of participants with previously undiagnosed HIV, HBV, HCV, syphilis, diabetes and hypertension who therefore benefited from more timely medical care.

Many countries internationally have been found to have high HIV prevalence among prisoners, with 10 low and middle income countries reporting >10% prevalence among prisoners [2], with rates often significantly higher when compared to national data. This study confirmed that the prevalence of HIV among prisoners in Mexico City was higher (0.7%) than the general population, and identified significant numbers of participants previously unaware of their status. The majority of those diagnosed through the study have subsequently been followed up and treated by a specialist HIV clinic in another prison in Mexico City. Clinic staff have reported that prisoners diagnosed through the study tended to have been diagnosed earlier than those diagnosed through the previous health service (when testing was based on clinical suspicion). They have also described favorable outcomes in terms of maintenance on treatment with low default rates and improved morbidity and mortality. However, we suspect that our results still represent an underestimation of the true prevalence of HIV. The majority of male prisoners previously known to have HIV were cared for in a Centre not included in the study (about 80 prisoners living with HIV received care in this other Centre at the start of the project). Additionally, field staff anecdotally reported that most of transgender prisoners declined to participate in the study, citing stigmatization as the predominant reason. They are a particularly vulnerable population with a high risk of transmissible infections, particularly HIV. A subsequent targeted study conducted by our team found that the prevalence of HIV among transgender prisoners in 3 Mexico City’s prisons, including the 2 male prisons in this study was 31% [26]. These results were not included in this analysis, representing another source of underestimation of HIV prevalence in our study and therefore difficulty in determining exactly how great the increased risk compared to the general population.

The prevalence of HCV and Anti-TP+/VDRL+ antibodies was also greater for both sexes compared with national data. Our analysis suggests significantly higher odds of these infections among the prison population than the general population.

There are many reasons for the increased prevalence of transmissible infections in prisoners. Behavior and circumstances before imprisonment, such as injecting drug use, sexual behavior, including sex work, exposure to inter-personal violence and poverty, increase the risks of infections prior to imprisonment. But there is also the risk of transmission within the prison. Despite prohibition, many studies have shown that injecting drug use continues, needle-sharing increases [27] and unsterile equipment is commonly used for tattooing. These practices increases the risk of transmission of blood-borne infections, particularly HCV, but also HBV and HIV [7,28].

The analysis of behavioral characteristics from the questionnaire revealed that participants reported high levels of drug use and unsafe tattooing both before and during imprisonment. These factors are likely to partly explain the relative increase in transmissible infections compared to the general population. Even though drug use generally decreased during imprisonment, significant numbers of prisoners who had not used drugs in the 6 months prior to incarceration reported using drugs in prison, indicating that prison may be a risk factor for drug use among individuals who may not have otherwise used them. Reported needle sharing for drug use continued within the prison (though less frequently than prior to imprisonment) and needle sharing among those injecting non-prescribed vitamins increased. In combination with using unsafe tattooing equipment within the prison, these represent a potential risk for incident infections within prison and the need to consider the use of injecting /tattooing equipment exchange programs.

Sexual transmission of diseases within prisons is less well understood. In Mexico City prisons some prisoners are allowed conjugal visits. Despite this, unprotected sex between prisoners or with custodial staff may facilitate transmission of STIs, which if untreated can lead to further spread within the prison and the wider community on release, including through the disruption of stable relationships [29,30]. Though the frequency of sex in prison was not assessed in this analysis, reported rates of condom use in prison were low, which indicates the need to both provide condoms and educate about the importance of their use. High rates of sex work and abuse before imprisonment will require more complex interventions to reduce risk.

On comparing chronic health problems with national statistics for the same age groups, the prevalence of obesity was found to be similar for female but lower for male participants. The explanation for this is not clear and warrants further investigation, but may be related to increased physical activity—both due to exercise and labor, among male prisoners. Previously diagnosed diabetes and hypertension was lower in men compared to women, though this varied slightly with age, and may be linked to the differences in levels of obesity. Previously undiagnosed hypertension was lower than national prevalence however this is likely to reflect the lower average age of the study population, and national data by age was not available for comparison. Over the last three national health surveys (1993–2006), a marked increase has been seen in type 2 diabetes (6.7→14.4%), metabolic syndrome (26.6→36.8%), hypertension (23.8→30.7%) and hypercholesterolemia (27→43.6%) in the general population [31]. Even if the prevalence of these problems remains lower in the prison population, it will still constitute a significant need for intervention as current strategies pay little attention to the detection and management of chronic health problems in prison. This may be reflected in the inadequacy of blood pressure control for the majority of participants that had previously been diagnosed with hypertension. In addition, smoking was much higher than the general population which may lead to increased chronic health problems for these prisoners in the future. The need to put chronic health problems in prisons on the agenda has been recognized by expert organizations such as the American Diabetes Association (ADA) who have developed specific guidelines for early detection and treatment of diabetes in prisoners [32]. Guidelines should be developed or adopted for chronic health problems in Mexican prisons.

In addition to those already mentioned, this study has several important limitations. The cross-sectional design allowed for prevalence data to be calculated, but was unable to assess the incidence of the health problems studied or infer causality. While health screening was widely acceptable among prisoners, a lower percentage of men participated than women. It was not possible to perform differences analysis because of a lack of information for the non-participants group, but the difference may reflect that the male prisons are much larger facilities and, in addition to those who may not have wished to participate due to perceived stigma if results were positive, it was sometimes difficult to access prisoners who were confined for custodial reasons. As those with positive results were referred on for further treatment, details of uptake or success of treatment were not available. Further investigation is warranted for other conditions, particularly for tuberculosis, where poor ventilation and hygiene, overcrowded cells and insufficient treatment have led to outbreaks in several countries and much higher prevalence than in the community [33]. Further assessment is also particularly needed for mental health problems, which are often unidentified and much more prevalent in prisoners than the general population.

Due to funding constraints, we were not able to test the entire population for diabetes and dyslipidemia, but rather screened the population using a self-reported risk assessment to determine who would be tested. This process may have underestimated the true prevalence. Additionally, participants did not undergo more detailed assessment of HBV and HCV to confirm subjects currently infected, however the antibody prevalence to HCV reflecting viral exposition was measured with the same methodology as in the national study and therefore comparative data remains reliable.

The questionnaire sample was relatively small, and the response rate varied between questions—it was particularly low with respect to questions about sexual abuse—and due to the illegality of drug use in prison or sex between prisoners the data gleaned from this aspect of the study may therefore not provide a full picture of the socio-demographic/risk profile of Mexico City’s prisoners. Furthermore, questions regarding pre- and post-admission to prison behavior were framed with different time-spans—longer pre-admission to allow for greater capture of information and shorter during imprisonment to allow for comparison between those with different lengths of imprisonment. These data may also be subject to recall bias, making comparisons between behavior pre- and post-admission to prison difficult.

Despite these limitations, the data gathered from this study have already been useful for the participants who have benefited from more timely treatment and referral for specialist care. We also expect reduced long-term health system costs from averted complications and onward transmission of infections. The study also helped raise awareness of important health problems among prisoners and prison staff and helped promote the importance of a healthy diet and physical activity. Furthermore, despite its limitations, our study represents the first effort of this magnitude to rigorously document the health status and its determinants of prisoners in Mexico, and one of the first among low- and middle-income countries.

This data is the first step in developing future health prevention and treatment interventions. There are many challenges ahead. This study has reiterated the vulnerability of prisoners—with lower educational attainment and income than the general population, significant rates of smoking, drug use, physical and sexual violence, sex work and low rates of condom use. Many prisoners lacked access to good healthcare prior to imprisonment and, with increasing incarceration rates in Mexico City over the last decade without a significant change in the capacity or facilities, their access to health services in prisons has been limited and their living conditions have worsened. Policies must therefore be comprehensive and include those to reduce overcrowding and provide better access to clean water, sanitation and health services. Health education and screening should be performed on admission and at intervals; targeted strategies may be needed for higher risk groups such as transgender individuals. With more health problems identified, health services will need to be expanded and strengthened. Data from the HIV clinic has shown significant improvements in the health of those it cares for, but this is the only specialist clinic within the prison. Organizing release for prisoners to go to hospitals or specialist clinics is challenging and therefore further consideration is needed as to how to best provide care for those with complex problems, and will likely require both improvements in general health services within the prison and more collaboration with specialist services outside the prison.

## Conclusion

**T**his study was unique in its scope, providing a comprehensive diagnostic package to prisoners in Mexico City. The prisoners involved in the study represent a vulnerable population both in terms of the higher levels of transmissible infections as well as education, poverty, experience of violence and risk behaviors, and will often have had poor access to healthcare in the past. The identification of significant numbers of prisoner health problems reinforces the need to address the rights of prisoners to healthcare, as well as the need to target prevention strategies that reflect the complex socio-demographic characteristics and risk behaviors of this population. To successfully address these challenges, strategies require collaborative work between prisoners, healthcare providers, prison officials and government, in addition to continued research to provide accurate data on which to prioritize policies and evaluate interventions.
